# Cardiomyocyte-restricted high-mobility group box 1 (HMGB1) deletion leads to small heart and glycolipid metabolic disorder through GR/PGC-1α signalling

**DOI:** 10.1038/s41420-020-00340-9

**Published:** 2020-10-20

**Authors:** Peng Yu, Ming Liu, Baoli Zhang, Ying Yu, Enyong Su, Shiyao Xie, Lei Zhang, Xue Yang, Hong Jiang, Ruizhen Chen, Yunzeng Zou, Junbo Ge

**Affiliations:** 1 Department of Endocrinology and Metabolism, Fudan Institute of Metabolic Diseases, Zhongshan Hospital, Fudan University, Shanghai, China; 2 Department of General Practice, Zhongshan Hospital, Shanghai Medical College of Fudan University, Shanghai, China; 3 Shanghai Institute of Cardiovascular Diseases, Shanghai Clinical Bioinformatics Research Institute, Zhongshan Hospital, Shanghai Medical College of Fudan University, Shanghai, China

**Keywords:** Developmental biology, Heart failure

## Abstract

Cardiac growth and remodelling are key biological processes influencing the physiological performance of the heart, and a previous study showed a critical role for intracellular HMGB1 in vitro. However, the in vivo study, which used conditional Hmgb1 ablation, did not show a significant effect on cellular or organic function. We have demonstrated the extracellular effect of HMGB1 as a pro-inflammatory molecule on cardiac remodelling. In this study, we found that HMGB1 deletion by cTnT-Cre in mouse hearts altered glucocorticoid receptor (GR) function and glycolipid metabolism, eventually leading to growth retardation, small heart and heart failure. The subcellular morphology did not show a significant change caused by HMGB1 knockout. The heart showed significant elevation of glycolysis, free fatty acid deposition and related enzyme changes. Transcriptomic analysis revealed a list of differentially expressed genes that coincide with glucocorticoid receptor function in neonatal mice and a significant increase in inflammatory genes in adult mice. Cardiac HMGB1 knockout led to a series of changes in PGC-1α, UCP3 and GyK, which were the cause of metabolic changes and further impacted cardiac function. Ckmm-Cre Hmgb1^fl/fl^ mice did not show a specific phenotype, which was consistent with the reported negative result of cardiomyocyte-specific Hmgb1 deletion via MHC-Cre. We concluded that HMGB1 plays essential roles in maintaining normal cardiac growth, and different phenotype from cardiac-specific HMGB1-deficient mice may be caused by the cross with mice of different Cre strains.

## Introduction

Cardiac growth and remodelling are key biological processes influencing the physiological performance of the heart. Cardiomyopathy is one of the most common causes of heart failure and is characterized by cardiac remodelling and contractile dysfunction. However, the molecular mechanisms underlying the pathophysiology of decompensated heart failure remain largely unknown. Strategies targeting new molecules or factors are urgently needed to prevent this transition under pathological conditions^1^.

High-mobility group box 1 (HMGB1), which exhibits diverse biological functions depending on cellular location, is a non-histone DNA-binding nuclear protein present in nearly all cell types^2^. Intracellular HMGB1 regulates autophagy, facilitates DNA repair and regulates transcriptional regulation^3–5^. However, in vivo validation of the versatile functions of HMGB1 shows its dispensable role in adult organisms^6^. Furthermore, newborn Hmgb1-deficient mice exhibited lethal hypoglycaemia^7,8^. Mice of different Cre strains could lead to diverse phenotypes resulting from asynchronous functional loss of the floxed genes, which may reveal more detailed molecular roles in biological processes^9^.

This work focused on the role of HMGB1 in the development of the heart by conditional cardiac-specific HMGB1 knockout using cTnT-Cre/+ mice, in which the target loxP sites could recombine starting from E7.5, resulting in earlier and more uniform recombination in the developing heart than that induced by Myh6-Cre^10–12^. We found that cardiac-specific knockout of HMGB1 resulted in small and dysfunctional hearts and the underlying mechanisms of glucocorticoid receptors (GRs) and metabolic changes. Our data showed that HMGB1 plays a vital role in shaping cardiac function.

## Results

### Generation of cardiac HMGB1 knockout mice

**Fig. 1:**
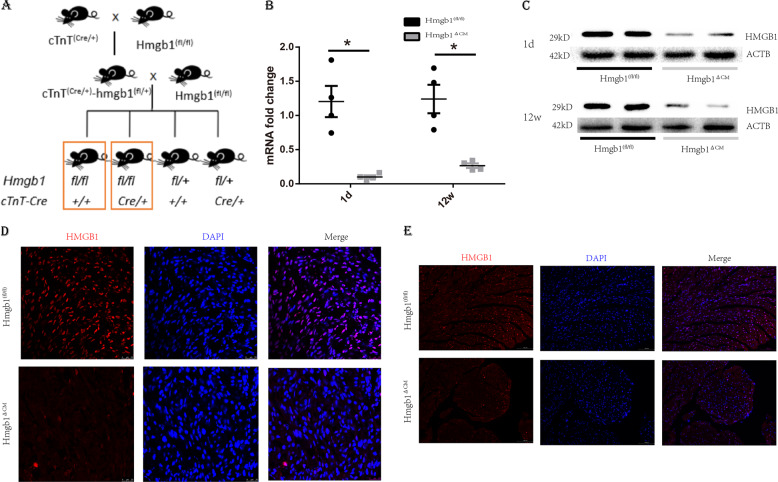
Generation and characterization of cardiac-specific HMGB1 knockout mice. **a** Schematic diagram of the mouse breeding strategy to generate cardiac-specific HMGB1 knockout mice. **b** The mRNA levels of the Hmgb1 gene in control and HMGB1^△CM^ hearts measured by quantitative RT-PCR at neonatal and 12-week-old. *n* = 4 mice per group. *p < 0.05 versus control, *t*-test, mean ± SEM. **c** Western blot analysis of HMGB1 protein in the whole-heart tissue of control and HMGB1^△CM^ hearts at neonatal and 12-week old. ACTB was used as the loading control for the whole heart. *n* = 4 mice per group. **d** Immunostaining of the heart at the neonatal stage, bar = 25 μm. **e** Immunostaining of the hearts of HMGB1 mice at 12-week-old, bar = 100 μm.

To investigate the roles of HMGB1 in cardiomyocytes in vivo, we generated cardiac-specific HMGB1 knockout mice using Cre-loxP technology. The mouse breeding strategy to generate cardiac-specific HMGB1 knockout mice is shown in Fig. 1a. Gel electrophoresis of PCR genotyping of Cre and Hmgb1-loxp is shown in Fig. S1a and 1b separately. Moreover, verification of the HMGB1 knockout was demonstrated by reverse transcriptase PCR (RT-PCR) with primers specific to exons 2–4 of HMGB1 (Fig. 1b). HMGB1 knockout was confirmed by western blotting demonstrating that both neonatal and 12-week-old Hmgb1^△CM^ mice had a significant decrease in HMGB1. (Fig. 1c) The expression of HMGB1 in heart tissue is reduced, but not abolished. The residual HMGB1 may result from stromal cells without cardiac Troponin T (cTnT) expression. We further exclude the leakage of the cTnT-Cre mice using western blot to detect HMGB1 in the liver, spleen, lung, kidney, muscle, ovary and testis, which showed no difference between the two groups (Fig. S1e). Immunofluorescence (IF) staining in frozen cardiac sections from neonatal and adult mice revealed undetectable HMGB1 expression in the Hmgb1^△CM^ group compared to the Hmgb1^fl/fl^ group (Fig. 1d, e).

### Genetic deletion of HMGB1 in cardiomyocytes results in growth retardation and small hearts

**Fig. 2:**
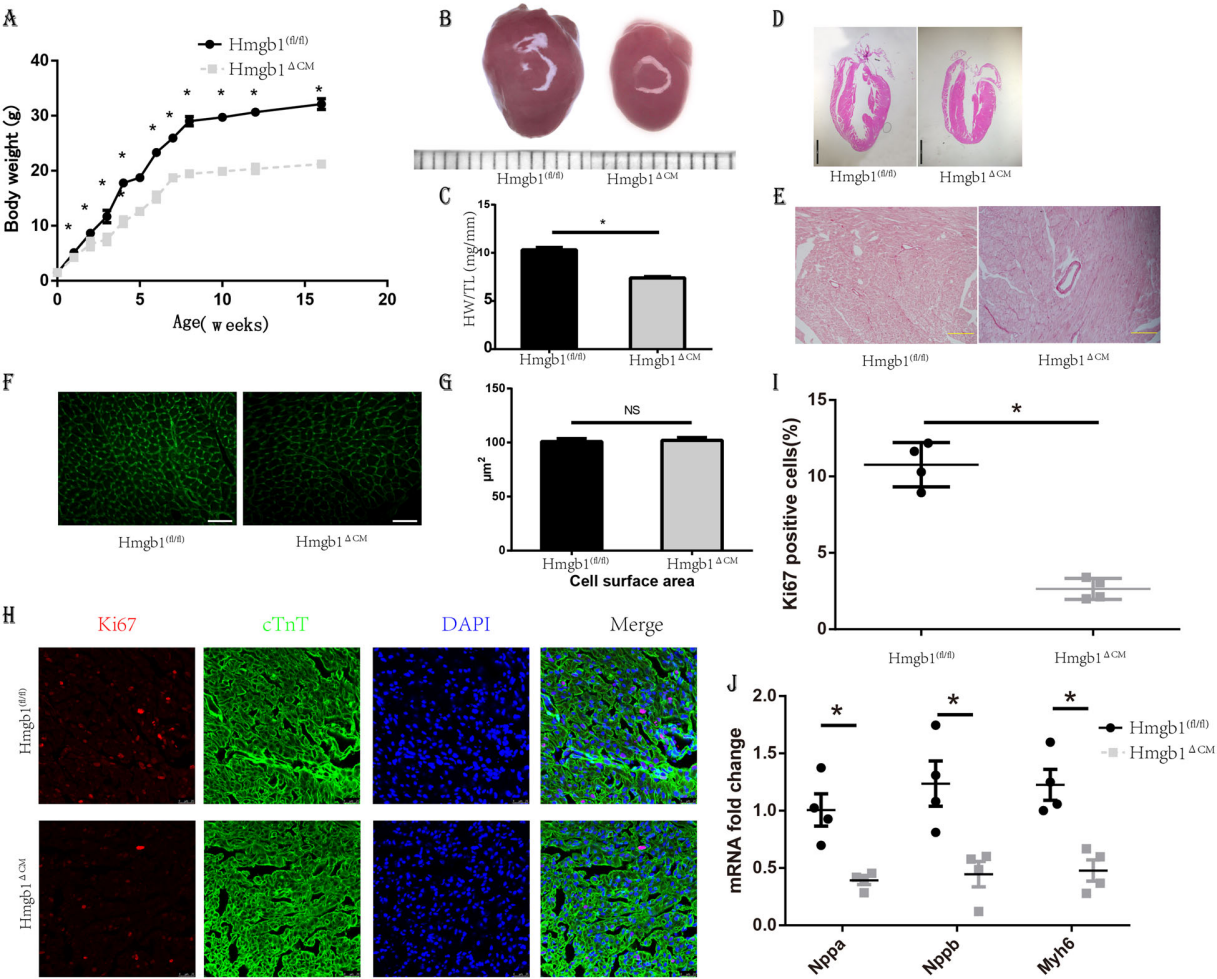
Deletion of cardiac HMGB1 results in mouse growth retardation and small hearts. **a** Body weight curves of control (*n* = 6) and HMGB1^△CM^ (*n* = 6) mice. **b** Representative photos of freshly excised whole hearts, 12-week-old. Grid = 1 mm. **c** Heart weight/tibia length (HW/TL) ratios, 12 weeks. **d** H&E-stained sections of control and HMGB1^△CM^ mice 12 weeks, bar = 2 mm, *n* = 6/group. **e** Sirius red staining of ventricular sections revealed Increased fibrosis (red) in HMGB1^△CM^ hearts at 12-week-old, bar = 100 μm, *n* = 6/group. **f** WGA staining, scale bar = 40 μm. **g** Cross-sectional area (CSA) of cardiomyocytes was quantitatively analysed and showed no significant difference at 12-week-old, *n* = 6/group. **h** Immunofluorescent staining showing the presence of proliferating Ki-67 + (red) and cTnT + (green) at 4-day-old, bar = 25 μm. **i** Bar charts showing the percentage of cardiomyocytes that are Ki-67+, *n* = 4/group. **j** qRT-PCR of canonical hypertrophic marker genes in neonatal mouse hearts, *n* = 6/group. *p < 0.05 versus control, *t*-test, mean ± SEM.

Hmgb1^△CM^ showed significant retardation of body growth (Fig. 2a). The female mice were infertile, while the males were fertile after 16 weeks of age, in comparison with the HMGB1^fl/fl^ mice, which could reproduce from 6–8 weeks old. We detected HMGB1 in ovary and testis to exclude the work of Cre in gonads which showed no difference (Fig. S1e). The littermates were born with similar appearance and body weight (Fig. S1c), whereas the Hmgb1^△CM^ littermates showed significantly decreased body mass (Fig. S1d).

We suspected that the retardation of body growth was caused by cardiac deficiency. We found that the gross appearance of Hmgb1^△CM^ hearts was diminished (Fig. 2b) and that heart weight/tibial length ratio (HW/TL) and heart weight/body weight ratio (HW/BW) were decreased significantly (Fig. 2c and Fig. S2a). No structural abnormalities were observed by haematoxylin and eosin (H&E) staining (Fig. 2d), and there were also no significant structural differences in the neonatal heart (Fig. S2b). Furthermore, Sirius red staining demonstrated that the degree of fibrosis in Hmgb1^△CM^ hearts was more severe (Fig. 2e). Wheat germ agglutinin (WGA) staining demonstrated no significant variance in the cell surface between the two groups (Fig. 2f, g). To detect the cause of small heart size, we performed staining with Ki-67, a cellular marker for proliferation, which was significantly decreased in neonatal Hmgb1^△CM^ mice (Fig. 2h, i). Additionally, Hmgb1^△CM^ inhibited myocardial expression of canonical hypertrophic marker genes (Fig. 2j), and transmission electron microscopy (TEM) showed no mitochondrial structural changes in the Hmgb1^△CM^ mice (Fig. S2c).

### Cardiac HMGB1 knockout mice developed cardiac dysfunction

**Fig. 3:**
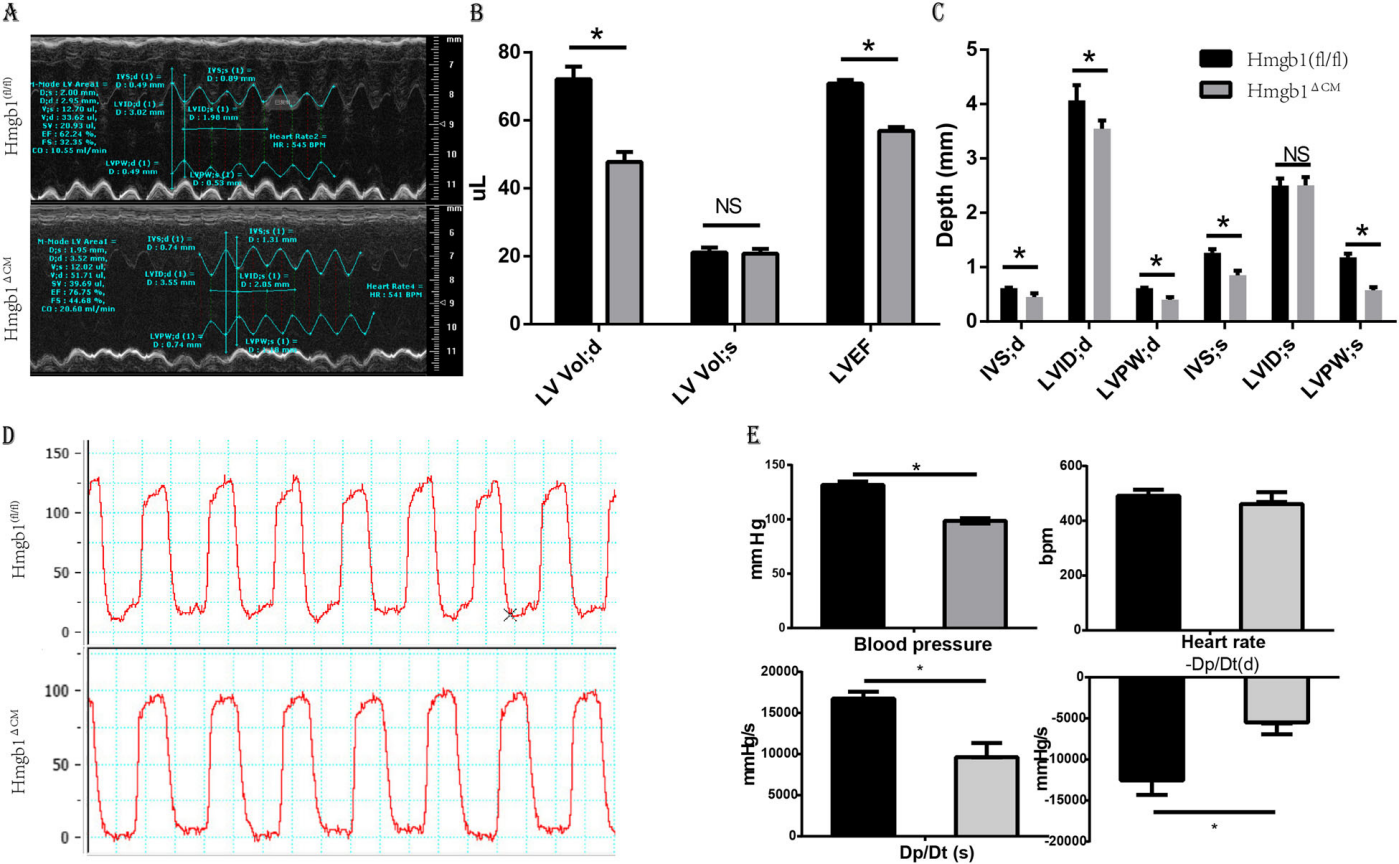
Assessment of adult cardiac morphology and function by echocardiography and invasive haemodynamic analysis. **a** Representative left ventricular M-mode echocardiographic tracings. **b** Measurement of left ventricular volume at end-diastole (LV Vol;d) and at end-systole (LV Vol;s), and left ventricular ejection fraction (EF). **c** Measurement of left ventricular internal diameter at end-diastole (LVID;d) and at end-systole (LVID;s), interventricular septal wall thickness at end-diastole (IVS;d) and at end-systole (IVS;s), and left ventricular posterior wall thickness at end-diastole (LVPW;d) and at end-systole (LVPW;s), *n* = 6/group. **d** Invasive haemodynamic analysis of blood pressure. **e** Blood pressure, heart rate, d*p*/d*t*max and −d*p*/d*t*max in mice, *n* = 6/group. *p < 0.05 versus control, *t*-test, mean ± SEM.

We further performed functional and morphological examinations of the heart. Echocardiographic measurements indicated compromised cardiac function (Fig. 3a). In addition to the thinner ventricular wall and smaller ventricular volume (Fig. 3b, c), the Hmgb1^△CM^ mice showed reduced left ventricular ejection fraction (LVEF) and fractional shortening (Table 1). Haemodynamic parameters showed that HMGB1-cKO induced a significant decrease in LV peak systolic pressure (LVP sys, 99 mmHg) compared to the HMGB1^fl/fl^ condition (131 mmHg) (Fig. 3d). Moreover, both d*P*/d*t*max (peak rate of ventricular pressure rise) and d*P*/d*t*min (peak rate of ventricular pressure decline) were decreased significantly by cardiac-specific knockout of HMGB1 (Fig. 3e).

**Table 1:** Echocardiographic parameters of the two groups.

	Hmgb1 (fl/fl)	Hmgb1^△CM^	*p* value
IVS;d	0.61 ± 0.022	0.49 ± 0.014	0.0001
LVID;d	4.037 ± 0.092	3.393 ± 0.088	0.0001
LVPW;d	0.621 ± 0.019	0.465 ± 0.015	0.0001
IVS;s	1.232 ± 0.029	0.854 ± 0.017	0.0001
LVID;s	2.431 ± 0.071	2.4 ± 0.071	0.77
LVPW;s	1.146 ± 0.029	0.68 ± 0.036	0.0001
LV Vol;d	72.01 ± 3.80	47.80 ± 2.89	0.0001
LV Vol;s	21.09 ± 1.50	20.74 ± 1.44	0.87
%EF	70.86 ± 1.07	56.93 ± 1.09	0.0001
% FS	39.83 ± 0.90	29.08 ± 0.70	0.0001
Heart rate	521.4 ± 5.37	508.8 ± 5.46	0.13

*IVS;d* interventricular septal wall thickness at end-diastole, *IVS;s* interventricular septal wall thickness at end-systole, *LVID;d* left ventricular internal diameter at end-diastole, *LVID;s* left ventricular internal diameterat end-systole, *LVPW;d* left ventricular posterior wall thickness at end-diastole, *LVPW;s* left ventricular posterior wall thickness at end-systole, *LV Vol;d* left ventricular volume at end-diastole, *LV Vol;s* left ventricular volume at end-systole, *LVEF* left ventricular ejection fraction, *FS* fractional shortening.

### Cardiac HMGB1 knockout changed glucose and lipid metabolism

**Fig. 4:**
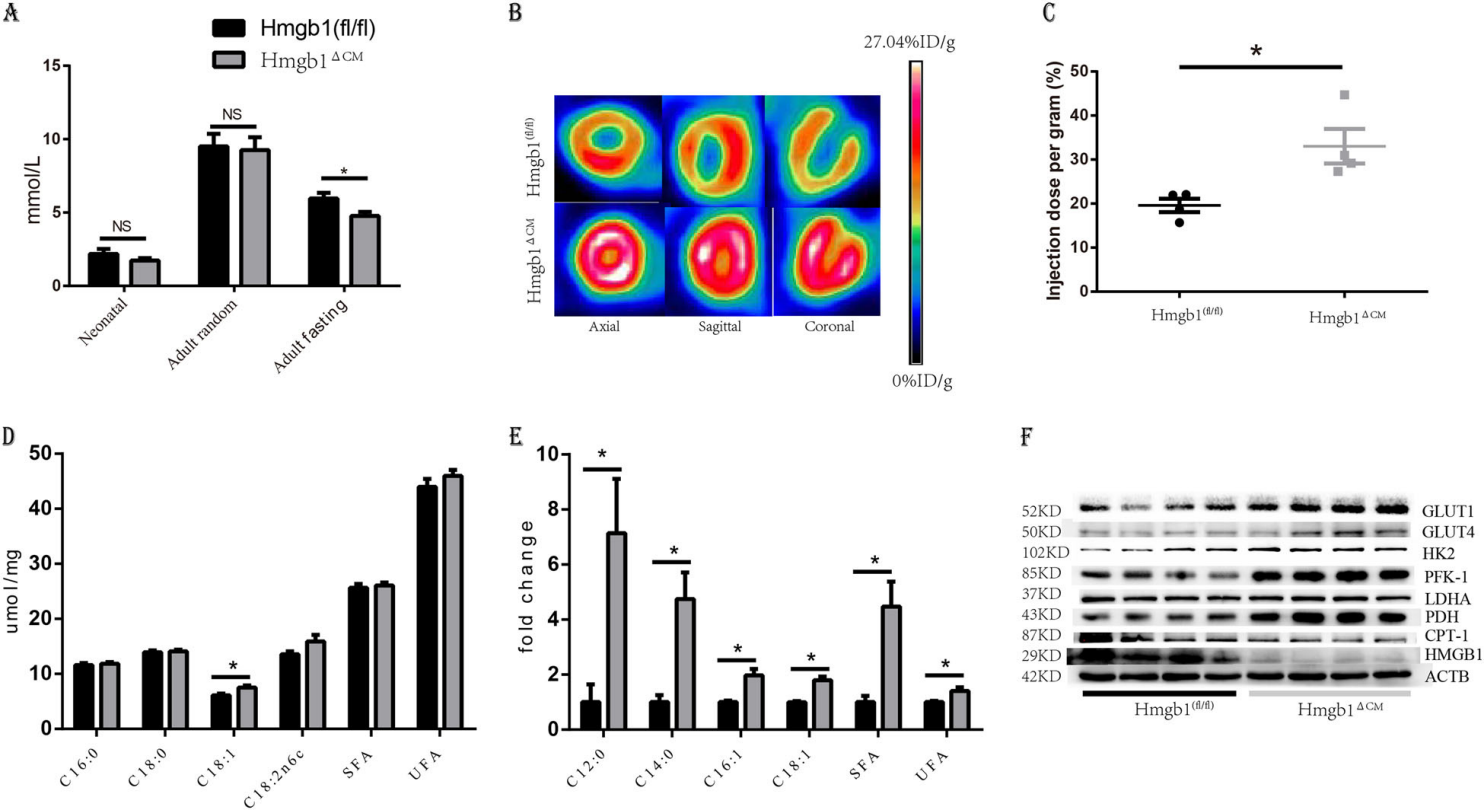
HMGB1 knockout switches cardiomyocytes to glycolytic metabolism. **a** Plasma glucose of control (*n* = 6) and HMGB1^△CM^ mice at neonatal and 12-week-old. **b** Representative 18F‐FDG images with micro-PET in the axial, sagittal and coronal planes in control (*n* = 4) and HMGB1^△CM^ mice at 12-week-old. **c** Myocardial FDG uptake measured as the standard uptake value (SUV); *n* = 4/group. **d** Quantitative analysis using GC-FID/MS of fatty acid composition in heart tissue from control or HMGB1^△CM^ mice at 12-week-old, *n* = 6/group. **e** GC-FID/MS analysis of free fatty acids in heart tissue from control or HMGB1^△CM^ mice at 12-week-old, *n* = 6/group. **f** Proteins relative to glycolysis and lipid metabolism expression in LV tissue normalized to ACTB at 12-week-old, *n* = 6/group. *p < 0.05 versus control, *t*-test, mean ± SEM.

As HMGB1^−/−^ mice have been reported to die because of severe hypoglycaemia^8^, we next analysed the blood glucose levels of newborns. At birth, the level of blood glucose showed a decreasing trend in the Hmgb1^△CM^ siblings. The random blood glucose measurements were similar for the adult mice. However, cardiac-specific HMGB1 knockout led to a significant decrease in fasting blood glucose (Fig. 4a). To further prove the HMGB1-dependent reconfiguration of energy metabolism, we performed an [18F]-fluorodeoxyglucose positron emission tomographic analysis in living animals to unambiguously demonstrate that HMGB1 affects myocardial metabolism in the beating heart. Our measurements revealed a significant increase in glucose uptake and glycolytic flux in the heart after deletion of HMGB1 in adult cardiomyocytes (Fig. 4b, c).

We further detected the total fatty acids and free fatty acids, and the total fatty acids between the two groups showed little difference (Fig. 4d). However, the cardiac-specific knockout of HMGB1 led to a significant elevation of free fatty acids (Fig. 4e), which might be caused by a disturbance in cardiac metabolism. We detected the enzymes involved in glucose and free fatty acid metabolism. The results showed an increase in the enzymes facilitating glycolysis, including GLUT1, GLUT4, HK and PDH, and a decrease in CPT1, a key enzyme involved in the utilization of free fatty acids (Fig. 4f).

### Cardiac HMGB1 knockout represses the glucocorticoid response

**Fig. 5:**
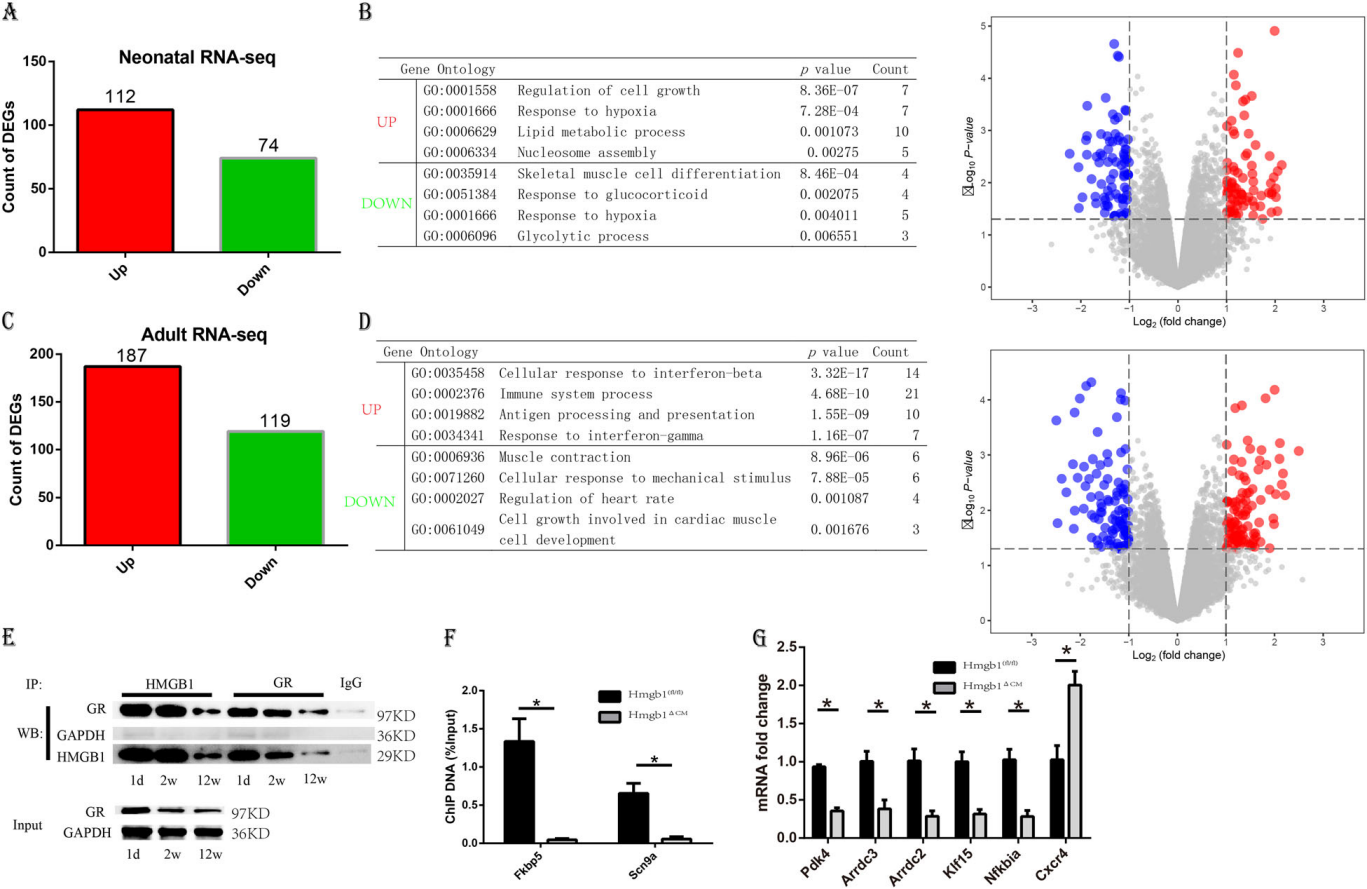
Global gene expression profile in neonatal and 12-week-old control and HMGB1^△CM^ hearts. **a** Total number of genes differentially expressed in the hearts of neonatal HMGB1^△CM^ compared to their control littermates. **b** Volcano plots and biological process most significantly associated with the dysregulated genes in the neonatal knockout hearts as determined by DAVID. **c** Total number of genes differentially expressed in the hearts of 12-week-old HMGB1^△CM^ mice compared to their control littermates. **d** Volcano plots and biological process most significantly associated with the dysregulated genes in the adult knockout hearts as determined by DAVID. **e** The physical interaction of HMGB1 and glucocorticoid receptor was analysed by immunoprecipitation and western blot analysis. **f** The effect of HMGB1 knockout on the recruitment of GR onto the Fkbp5 and Klf15 promoters was assessed by ChIP assays using an anti-GR antibody and neonatal hearts. Amounts of ChIP DNA were measured with quantitative PCR. *n* = 6/group. **g** qRT-PCR results of the GR downstream genes in neonatal hearts of the two groups, *n* = 6/group. *p < 0.05 versus control, *t*-test, mean ± SEM.

To gain further mechanistic insights into the function of HMGB1 in the heart, we performed RNA-sequencing analysis of hearts from Hmgb1^△CM^ and Hmgb1^fl/fl^ mice. The genes detected was shown in Tables S2 and S3. As expected, we detected numerous deregulated genes that reflect pathological changes in HMGB1-deficient hearts (Fig. 5a, c). More interestingly, gene ontology term analysis in Hmgb1^△CM^ hearts using the Database for Annotation, Visualization and Integrated Discovery (DAVID) revealed significant changes in genes involved in cell growth and response to glucocorticoids as well as changes in genes involved in metabolic processes (Fig. 5b, d). We hypothesized that HMGB1 knockout suppresses glucocorticoid pathways, further resulting in the adult phenotype, which includes metabolic changes and inflammatory activation.

Glucocorticoids work through multiple mechanisms to bring about their desired effects^13^. HMGB1 has been implied to be a cofactor to facilitate the effects of GRs^3,8,14^. We therefore reasoned that HMGB1 physically interacts with GRs and prepared WT mouse heart tissue lysates from neonatal, 2-week-old and 12-week-old mice for immunoprecipitation using anti-HMGB1 or GR antibodies. HMGB1 pulled down GR, and reciprocal immunoprecipitation of GR pulled down HMGB1 (Fig. 5e). Interestingly, Agresti et al. reported that HMGB1 facilitated GR binding with chromatin. Therefore, we performed chromatin immunoprecipitation (ChIP) in WT and Hmgb1^△CM^ neonatal mice, which showed that a lack of HMGB1 resulted in clearly diminished binding of GR to the genomic loci of Fkbp5 and Scn9a reported to be the GR-binding target (Fig. 5f)^15^. We further detected the downstream genes of GR from neonatal heart tissue^16^, and found the Pdk4, Arrdc3, Arrdc2, Abra, Klf15 and Nfkbia downregulated, Cxcr4 upregulated (Fig. 5g). The genes were related with glucose/lipid metabolism or inflammatory response either.

HMGB1 mainly functions as an inflammatory mediator, and glucocorticoids are used as anti-inflammatory therapy. The transcriptome of the adult heart showed that inflammatory genes were elevated significantly in 12-week-old mice. Therefore, the metabolic changes in Hmgb1^△CM^ mice may indicate a sequence of inflammation or an effect of GR dysfunction.

### The cardiac metabolic change was caused by GR-PGC-1α signalling

**Fig. 6:**
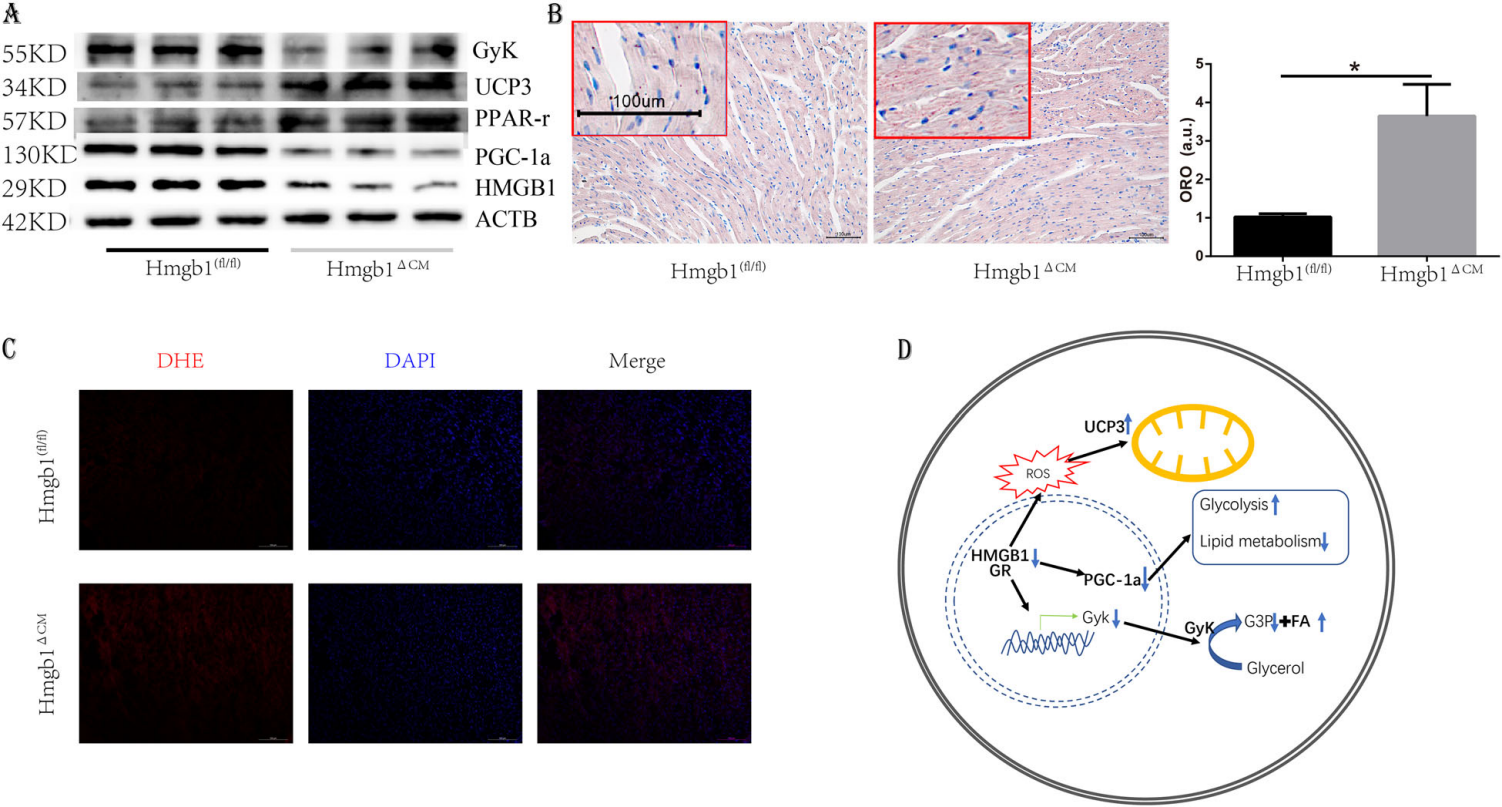
Deletion of HMGB1 in cardiomyocytes decreases PGC-1α signalling. **a** Western blot analysis and quantification of PGC-1α, PPAR-γ, UCP3 and GyK levels in control and HMGB1^△CM^ ventricles at 12 weeks (*n* = 6). **b** Representative heart Oil red O staining and quantification of control and HMGB1^△CM^ mice at 12 weeks, *n* = 6/group, bar = 100 μm. Insets from the images are magnified four times in order to highlight the lipid staining. Bar charts showing the percentage of cardiomyocytes that are oil red staining, a.u. arbitrary units, *p < 0.05 versus control, *t*-test, mean ± SEM. **c** Detection of superoxide by DHE staining at 12 weeks, bar = 100 μm, *n* = 4/group. **d** Model of the signalling mechanism: HMGB1 inhibits GR activity, further inactivates PGC-1α and GyK, causes increased glycolysis, and decreases lipid metabolism.

Physiological glucocorticoid levels improve the contractility of primary-mouse-foetal cardiomyocytes by inducing PGC-1α, a key mediator of glucocorticoid-induced maturation of cardiomyocytes^17^. Therefore, we detected PGC-1α and found downregulation of PGC-1α in Hmgb1^△CM^ mice. The downregulation of PGC-1α could impair fatty acid oxidation^18^ and increase glucose utilization^19^. UCP3 was also elevated in the heart and was postulated to export fatty acid anions from the mitochondrial matrix when fatty acyl-CoA levels were increased (Fig. 6a)^20^. Moreover, glycerol is a substrate for cardiac energy production and^21^ the glycerol kinase (GyK) could facilitate the glycerol oxidation and affect the deposition of fatty acid. We detected the GyK and found it decreased in the Hmgb1^△CM^ groups, which in line with the report that GyK deficiency cause growth retardation and fat metabolism alteration, as a downstream of PGC-1α^22,23^. Therefore, we proposed that HMGB1 deficiency may cause lipid deposition. Oil red O staining showed that HMGB1 knockout led to lipid deposition in the myocardium (Fig. 6b), which coincides with a recent study showing that ablation of Hmgb1 in intestinal epithelial cells causes intestinal lipid accumulation^24^. We further performed dihydroethidium staining (Fig. 6c) in the control and Hmgb1^△CM^ groups, which revealed that HMGB1 knockout resulted in elevated levels of ROS.

## Discussion

In our present study, we used a cardiac-specific cTnT-Cre system to delete HMGB1 in cardiomyocytes. We found that deletion of HMGB1 led to impaired heart growth and left ventricular dysfunction, accompanied by a dramatic decrease in body weight. This finding demonstrated that HMGB1 deletion in cardiomyocytes resulted in cardiomyopathy, which further led to heart failure and impaired body growth. Although knockout experiments using αMHC-Cre have shown that HMGB1 is dispensable for the heart^25^, the in vitro functions of HMGB1 have been demonstrated in mitochondrial quality control^26^. Our present study provided evidence for the first time, to our knowledge, that HMGB1 plays an essential role in maintaining normal cardiac growth and function in mice. On the other hand, deletion of HMGB1 in mouse cardiomyocytes was also associated with low blood glucose, and changes in the genes associated with inflammation and GRs were also consistent with previous studies showing that HMGB1 was associated with GR function^8^.

HMGB1 was first found to be a non-histone nuclear protein, facilitating nucleosome structure and enhancing DNA repair and chromatin modification^27^. Overexpression of HMGB1 could prevent DNA damage induced by pressure overload^5^. It was also reported to be essential for mitochondrial quality control or to facilitate mitochondrial restoration^28,29^. Previously, we showed that HMGB1 was involved in cardiac remodelling^5,30^. To investigate the effect of intracellular HMGB1 in the myocardium, we constructed cardiac conditional HMGB1 knockout mice and found that a lack of HMGB1 retarded heart growth and impaired its function, which may be caused by the effect on GR/PGC-1α and imbalance of glucose and fatty acid metabolism.

In our present study, we also performed RNA-sequencing analysis to identify the effects of HMGB1 deletion on cardiac gene homoeostasis. In the neonatal heart, there were 112 upregulated and 74 downregulated genes in the Hmgb1^△CM^ hearts relative to their control counterparts. Most of these genes are related to cell growth, muscle differentiation and the response to glucocorticoids, which further indicates the influence of genes involved in lipid metabolic changes. Therefore, we provided for the first time, at least to our knowledge, a list of genes affected by HMGB1-mediated neonatal mouse cardiomyocytes. In the adult mouse, HMGB1 knockout led to 187 upregulated and 119 downregulated genes. Interestingly, the upregulated genes are mostly related to the inflammatory process, in contrast with the opinion that HMGB1 acts as a pro-inflammatory protein. The downregulated genes are mostly related to cell muscle contraction or growth.

Conditional cardiac knockout of HMGB1 first led to decreased expression of cardiac foetal genes, including ANF and βMHC, suggesting that cardiac growth retardation was activated in mutant hearts caused by GR dysfunction and that abnormalities in cardiac morphology were not observed in mutant neonatal hearts, implying its critical physiological role. In addition, these findings indicate the emergence of abnormal morphology and reduced cardiac systolic function in mutant adult hearts. Later, the expression of inflammatory genes was increased in mutant hearts, likely as a compensatory mechanism or possibly a subsequent suppression of glucocorticoid effects.

It has been demonstrated that HMGB1 was dispensable in heart using MHC-Cre. Therefore, we constructed cardiac-specific HMGB1 knockout mice using Ckmm-Cre Hmgb1^fl/fl^, which did not show a significant phenotype (Fig. S3). Because the onset of Ckmm-Cre, Myh6-Cre and cTnT-Cre work at different times, we speculate that this difference in Cre expression timing might account for the different phenotypes observed in these cardiac-specific knockout mice^31^. Therefore, caution should be exercised when interpreting the results of different conditional knockout mice.

Taken together, our results demonstrate that HMGB1 is required for maintaining normal cardiac morphology and function. Loss of HMGB1 in cardiomyocytes altered glucocorticoid effects, impaired metabolic processes, and eventually resulted in small heart size and heart failure.

## Materials and methods

### Animals

Hmgb1^fl/fl^ mice, in which the Hmgb1^loxP^ allele was created by inserting loxP sites within intron 1 and intron 4 flanking exons 2–4 of Hmgb1, were kindly gifted by Prof. Taniguchi^7^. Mice with Cre recombinase driven by the rat cTnT promoter were kindly granted by Jiao et al.^11^. The mice were interbred to generate cardiac-specific HMGB1 knockout mice (Hmgb1^△CM^, cTnT^Cre/+^-Hmgb1^fl/fl^). The control mice used in this study were Hmgb1^fl/fl^ mice without the introduction of Cre recombinase. Mice used for experiments were confirmed to be the desired genotype by standard genotyping techniques and used at the age of 1 day and 12 weeks. All mice were developed on a C57BL/6 genetic background. The sample size was ten for each group of different age or genotype, among which none was excluded. No randomization was used. The researchers were not blinded to the experiments. Animal protocols were approved by the Institutional Animal Care and Use Committee (IACUC protocol no. 2017-0007).

### Total RNA extraction and quantitative reverse transcriptase polymerase chain reaction

Total RNA was extracted from heart tissue using TRIzol (Invitrogen Corp.). mRNA for HMGB1 was quantified in triplicate by SYBR Green quantitative RT-PCR. The PCR mixture was prepared using SYBR Green Master Mix (TaKaRa, Dalian, China), and the primers used are listed in Supplementary Table S1.

### mRNA library construction and RNA-sequencing

Oligo(dT)-attached magnetic beads were used to purified mRNA. Purified mRNA was fragmented into small pieces with fragment buffer. Then first-strand cDNA was generated using random hexamer-primed reverse transcription, followed by a second-strand cDNA synthesis. The cDNA fragments were amplified by PCR, and products were purified by Ampure XP Beads, and then dissolved in EB solution. The double-stranded PCR products were heated, denatured, and circularized by the splint oligo sequence to get the final library. The final library was amplified with phi29 to make DNA nanoball (DNB), which were loaded into the patterned nanoarray and single-end 50 bases reads were generated on a BGIseq500 platform (BGI-Shenzhen, China)^32^.

RNA-sequencing was used to transcriptome profiling^33^. Bowtie was used for aligning short DNA sequence reads to large genomes^34^. And we used RSEM for quantifying transcript abundances from the RNA-Seq data^35^. The differential expression genes (DEGs) were calculated using NOISeq^36^, and the differential expression probability more than 0.6 was considered significant. Then we analysed biological process of the DEGs using DAVID 6.8 (https://david.ncifcrf.gov/). The raw data of RNA-Sequencing were deposited at http://www.ncbi.nlm.nih.gov/bioproject/656926.

### Western blot analysis

Myocardial protein extraction and western blotting were performed as previously described^37^, using antibodies against the following proteins: GR (Thermo Fisher, MA1-510), HMGB1 (Abcam, ab18256), GLUT1 (CST, 12939), GLUT4 (CST, 2213), HK2 (CST, 2867), PFK-1 (Santa Cruz Biotechnology, sc-377346), LDHA (CST, 3582), PDH (CST, 3205), CPT-1B (Abcam, ab134988), GyK (Abcam, ab126599), UCP3 (Thermo Fisher, PA1-055), PPAR-γ (Abcam, ab45036) and PGC-1α (CST, 2178). Horseradish peroxidase-conjugated secondary antibody was used (Jackson, AB_10015289, AB_2313567). The band intensity of proteins of interest was normalized to that of β-actin (Bioworlde, AP0060).

### Co-immunoprecipitation experiments

HMGB1 or GR was captured from heart tissue using HMGB1 antibody or GR antibody. Mouse IgG antibodies (Santa Cruz Biotechnology sc-2025) and rabbit IgG antibodies (Santa Cruz Biotechnology, sc-2023) were used as a control for non-specific-binding proteins. Heart tissues were dissected, homogenized and further resuspended in lysis buffer (Beyotime P1003J). After incubation on ice and centrifugation, the supernatant was quantified by Bradford assay, and 1 mg of lysate was immediately precleared with Protein A/G PLUS-Agarose (Santa Cruz Biotechnology, sc-2003) at 4 °C for 1 h. The precleared lysate was then coimmunoprecipitated overnight on a rotating wheel at 4 °C with anti-HMGB1 or GR antibody covalently coupled to Protein A/G PLUS-Agarose. For the control, the same amount of protein extract was incubated with normal mouse or rabbit non-specific IgG covalently coupled with Agarose. After the last centrifugation step, protein complexes were eluted from the beads at 95 °C for 5 min^38^.

### Chromatin immunoprecipitation

ChIP was performed using a SimpleChIP® Enzymatic Chromatin IP Kit (Agarose Beads) (Cell Signaling Technology). ChIP assays used the GR antibody (Thermo Fisher Scientific, MA1-510), positive control or normal IgG contained in the kit, and were performed according to the kit protocol. Primers used for qPCR analysis of ChIP experiments are listed in Table S1^15^.

### Echocardiography

Mice underwent transthoracic echocardiography using a high-frequency ultrasound system Vevo770 (VisualSonics, Toronto, Canada). During the echocardiographic study, each mouse was positioned on a heating pad to maintain normothermia and anaesthetized with isoflurane (3% for induction). Left ventricular structure and function were assessed, including left ventricular posterior wall end-diastolic and end-systolic thickness, left ventricular end-diastolic and end-systolic dimensions, and LVEF.

### Invasive haemodynamic study

Left ventricular haemodynamics were evaluated after echocardiography, as described previously^39,30^. Briefly, a micromanometer (Millar 1.4F, SPR 835; Millar Instruments, Houston, USA) was inserted into the right common carotid artery and carefully advanced into the left ventricle. The micromanometer was connected to a Power Laboratory System (AD Instruments, Castle Hill, Australia) to record heart rate and left ventricular end-systolic and end-diastolic pressure.

### Histological examination

Hearts were arrested after anaesthesia by ketamine and rapidly immersed in 4% neutral formaldehyde or optimal cutting temperature compound (OCT) compound. Neutral lipid accumulation in tissues were detected and quantification using oil red staining^40^. Specimens embedded in paraffin were sectioned at a thickness of 5 μm and stained with H&E, Sirius Red or WGA to assess myocardial morphology and cardiac collagen content, respectively. Images were analysed using Image-Pro Plus software (Media Cybernetics, Rockville, USA).

### Immunohistochemistry

For immunostaining, as shown previously^37^, paraformaldehyde (PFA)-fixed cryostat sections were rehydrated in phosphate-buffered saline (PBS) and blocked for 30 min in PBS containing 5% BSA and 2% nonimmune serum solution at room temperature. Sections were probed with HMGB1 (Abcam, ab18256), Ki-67 (Abcam, ab16667) and cTnT (BD Biosciences, 564767) (1:150 dilution) in blocking buffer overnight at 4 °C. After incubation, sections were washed three times with PBS and incubated with goat anti-rabbit or mouse IgG antibody (1:200; Invitrogen, A32732 or A32723) diluted in blocking buffer at room temperature for 2 h. Quantitative analysis of the positive areas was performed using ImageJ software.

### TEM examination

Briefly, freshly dissected left ventricular tissue was fixed with 2.5% glutaraldehyde (pH 7.4) for at least 2 h. After three washes in 0.1 M phosphate buffer followed by fixation with 1% osmium tetroxide, specimens were dehydrated in ethanol. Ultrathin sections (50–60 nm) were cut on an ultramicrotome (Leica, Wetzlar, Germany) and stained with 3% uranyl acetate and lead citrate. Images were acquired with a CM-120 microscope (Philips, Amsterdam, the Netherlands).

### Positron emission tomography (PET)/computed tomography (CT) imaging

For micro-PET/CT analysis, mice that had fasted overnight were anaesthetized with 5% isoflurane and intravenously injected with fluorodeoxyglucose (18F-FDG) prior to imaging; mice were scanned 45 min later (Inveon mPET/CT; Siemens, Knoxville, TN, USA). Image quality was improved by micro-CT anatomical alignment. Data were reconstructed with the three-dimensional ordered subset expectation maximization algorithm. The maximum standard uptake value (SUVmax) was determined as a measure of cardiac glucose uptake capacity, which was normalized to the body weight of mice and total injection dosage.

### Statistical analysis

Continuous variables are presented as the mean ± standard error of the mean. Intergroup differences were assessed by Student’s *t*-test or rank-sum test when non-normal distribution via STATA software 12.0. A *p* value of <0.05 was regarded as significant.

## Supplementary information

Figure S1

Figure S2

Figure S3

Table S1

Table S2

Table S3
