# Supplementary figures and images for: Cardiomyocyte-restricted high-mobility group box 1 (HMGB1) deletion leads to small heart and glycolipid metabolic disorder through GR/PGC-1α signalling
Source: Cell Death Discov. 2020 Oct 20;6:106. doi: 10.1038/s41420-020-00340-9 (PMC7575537; doi:10.1038/s41420-020-00340-9)

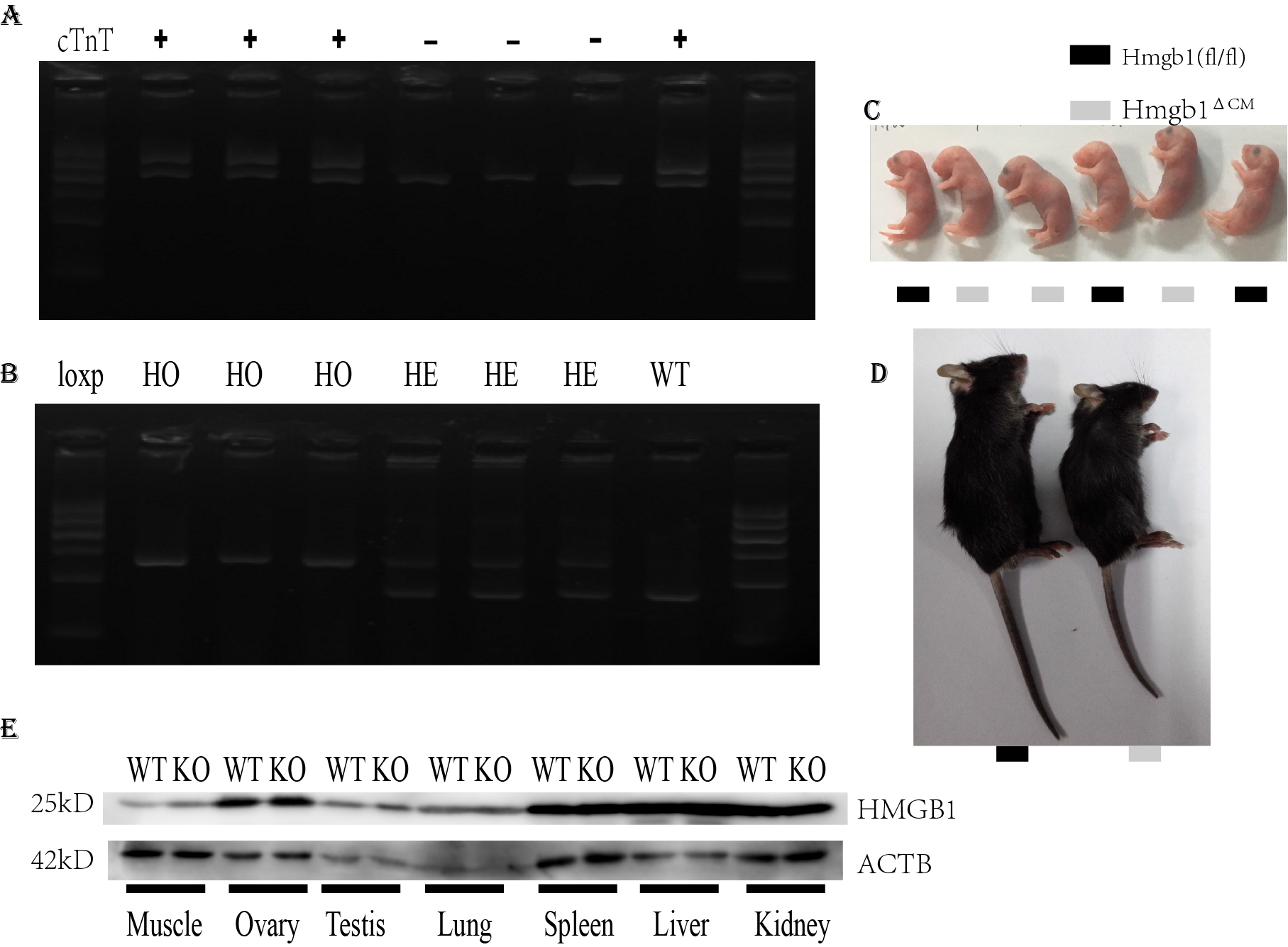

Supplement: Supplementary file 1 — Figure S1 [file 41420_2020_340_MOESM1_ESM.png]

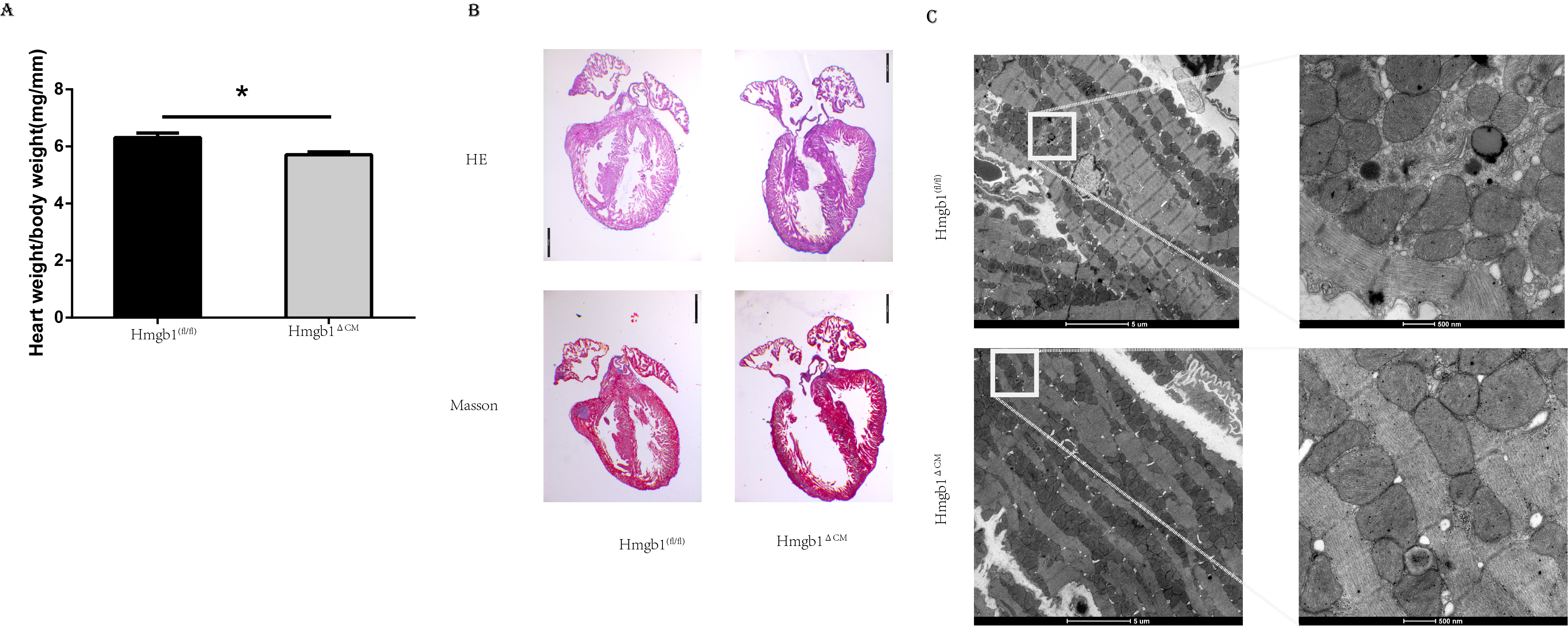

Supplement: Supplementary file 2 — Figure S2 [file 41420_2020_340_MOESM2_ESM.png]

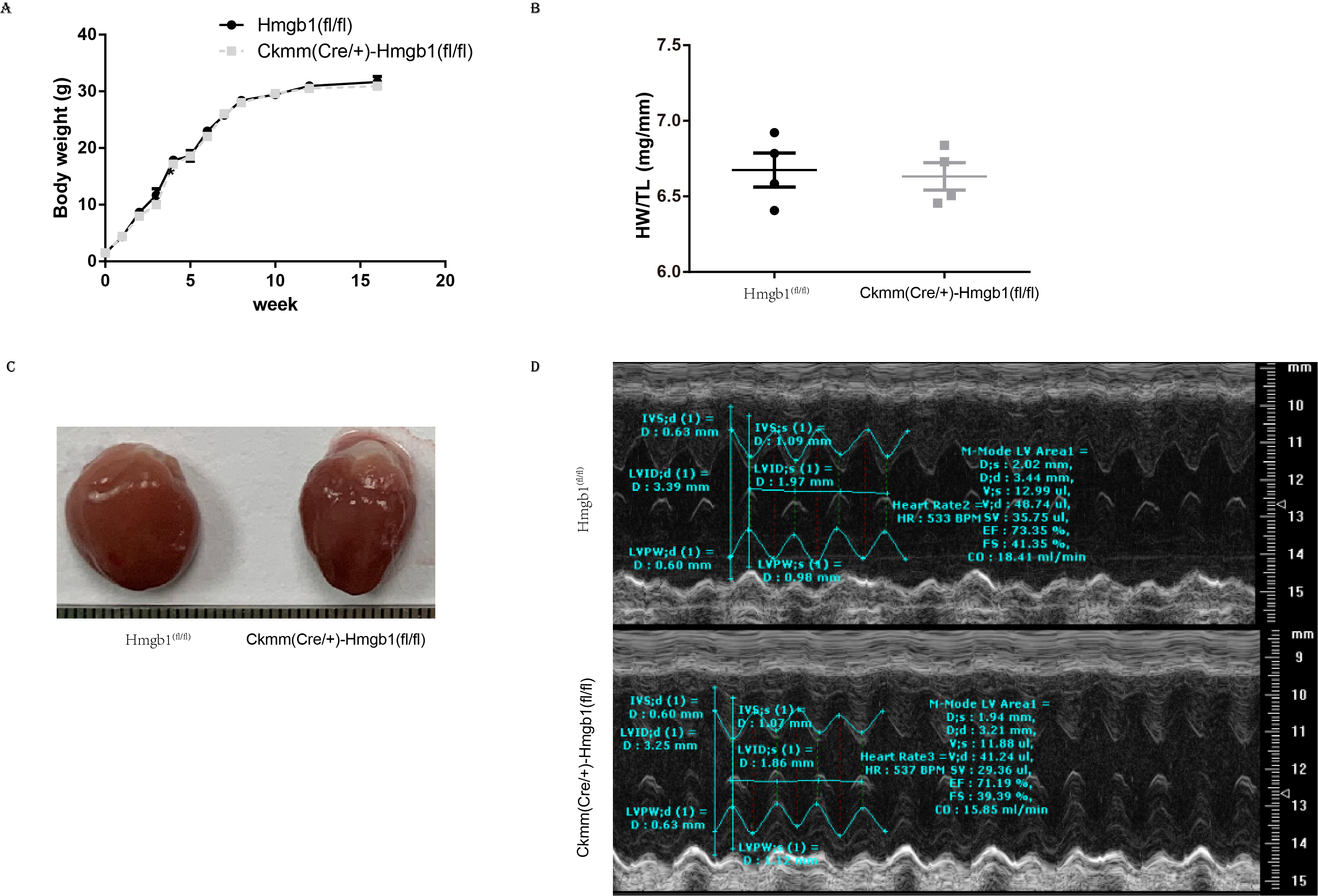

Supplement: Supplementary file 3 — Figure S3 [file 41420_2020_340_MOESM3_ESM.png]
