# Supplementary material for: Cardiomyocyte-restricted high-mobility group box 1 (HMGB1) deletion leads to small heart and glycolipid metabolic disorder through GR/PGC-1α signalling
Source: Cell Death Discov. 2020 Oct 20;6:106. doi: 10.1038/s41420-020-00340-9 (PMC7575537; doi:10.1038/s41420-020-00340-9)
Supplement: Supplementary file 4 — Table S1 [file 41420_2020_340_MOESM4_ESM.docx]

Table S1 qPCR primers used in this study (all target mouse)

| Genes | Forward | Reverse |
| --- | --- | --- |
| Hmgb1 | 5’-CGCTGGCTGGAGAGTAATGTT-3’ | 5’-AACGAGCCTTGTCAGCCTTT-3’ |
| Actb | 5’-GGCTGTATTCCCCTCCATCG-3’ | 5’-CCAGTTGGTAACAATGCCATGT-3’ |
| Fkbp5 | 5’-TCTGGTCACTGCAGACATCAC-3’ | 5’-GTCAGCACATCGAGTTCATGTG-3’ |
| Scn9a | 5’-GGCAGAAAAGAAACCAACATCC-3’ | 5’-TCTTAGACACAGTGCCTGCTG-3’ |
| Gapdh | 5’-GGGCGCCCTTCCAATTTTATC-3’ | 5’-TCACTTGTTGGACAGCACTG-3’ |
| CTnT-Cre | 5’-GGCTTAAAGGCTAACCTGGTGTG-3’ | 5’-GGAGCGGGAGAAATGGATATG-3’ |
|  | 5’-TTCTGGCGTCTGCTTTATCGGGATT-3’ | 5’-TCTTGCGAACCTCATCACTCGTTG-3’ |
| Nppb | 5’-GAGGTCACTCCTATCCTCTGG-3’ | 5’-GCCATTTCCTCCGACTTTTCTC-3’ |
| Myh6 | 5’-GCCCAGTACCTCCGAAAGTC-3’ | 5’-GCCTTAACATACTCCTCCTTGTC-3’ |
| Nppa | 5’-GCTTCCAGGCCATATTGGAG-3’ | 5’-GGGGGCATGACCTCATCTT-3’ |
| Pdk4 | 5’-AGCCGGCTGTCTTTACTGTTT-3’ | 5’-AAACCCATGATGGCTGGGAAA-3’ |
| Arrdc3 | 5’-AGGGTTTGTGCTCCCATAGC-3’ | 5’-ATGGTAGTGAGTGCCCAAGG-3’ |
| Arrdc2 | 5’-CTTTTCTGGGGTCCCGTTCC-3’ | 5’-TACACACACAAGCGGGACAA-3’ |
| Klf15 | 5’-TCAGTGTGACTTTGCTGTCA-3’ | 5’-GGTGGTGGATTCTACACGCA-3’ |
| Nfkbia | 5’-AGGTGATTTTCCAGAACCTAATGA-3’ | 5’-CAAGAAGGCGACACAGACCT-3’ |
| Cxcr4 | 5’-TGTTGCCATGGAACCGATCA-3’ | 5’-ACGCTGCTGTAGAGGTTGAC-3’ |
